# Improving the outcomes of primary care attenders with common mental disorders in developing countries: a cluster randomized controlled trial of a collaborative stepped care intervention in Goa, India

**DOI:** 10.1186/1745-6215-9-4

**Published:** 2008-01-25

**Authors:** Vikram H Patel, Betty R Kirkwood, Sulochana Pednekar, Ricardo Araya, Michael King, Daniel Chisholm, Gregory Simon, Helen Weiss

**Affiliations:** 1 Department of Epidemiology and Population Health, London School of Hygiene & Tropical Medicine, London, UK; 2 Research Program, Sangath, Goa, India; 3 Department of Psychiatry, University of Bristol, Bristol, UK; 4 Department of Psychiatry, Royal Free and University College Medical School, London, UK; 5 Department of Evidence and Information for Policy, WHO, Geneva, Switzerland; 6 Center for Health Studies, Group Health Cooperative, Seattle, USA

## Abstract

**Background and Objective:**

Common mental disorders (CMD) are a leading global burden of disease. Up to 30% of primary care attenders suffer from these disorders but most do not receive evidence-based drug or psychological treatments. There are no trials of interventions which attempt to integrate these treatments into routine primary care in developing countries. The aims of this trial (the MANAS Project) are to evaluate the clinical and cost-effectiveness of a collaborative stepped-care intervention for the treatment of CMD in India.

**Study Design:**

A cluster randomized controlled trial will be implemented in the state of Goa, on the west coast of India. Twenty-four primary care facilities, 12 from the government sector and 12 from the private sector, will be enrolled in two consecutive phases. For each sector, facilities will be randomly allocated within strata defined by urban/rural location, population size and presence of a visiting psychiatrist. Facilities will be randomly allocated to receive the collaborative stepped care intervention or the enhanced usual care control intervention. Both arms share two components of the intervention, viz., routine screening, and in the government clinics provision of antidepressants. In addition, the collaborative stepped care arm also provides a range of psychosocial treatments delivered by a specially trained Health Counselor, and supervision by a visiting Psychiatrist. A total of 3600 primary care attenders who are detected to suffer from a CMD based on a validated screening questionnaire will be recruited. The primary outcome is the proportion of subjects who recover from an ICD10 defined CMD at baseline by 6 months. Additional endpoints at 2 and 12 months will assess the speed and sustainability of achieving the primary outcomes. Other outcomes will include recovery from ICD10 defined depression and incidence of ICD-10 among individuals who were sub-threshold cases at baseline. Economic and disability outcomes will be assessed to estimate incremental cost-effectiveness ratios.

**Implications:**

This will be the first trial of the effectiveness of a complex intervention aiming to integrate efficacious treatments for CMD into routine primary care in a developing country. If effective, its findings will have relevance to policy makers who wish to scale up treatments for CMD in primary care across the world, but mostly in those countries where specialist mental health services are few.

**Study Registration:**

The MANAS project is registered through the National Institutes of Health sponsored clinical trials registry and has been assigned the identifier: NCT00446407

## Background

Common Mental Disorders (CMD) are depressive and anxiety disorders which are typically encountered in community and primary care settings [1]. Although depressive and anxiety disorders are classified as separate diagnostic categories in ICD10[2], the concept of CMD is valid for public health interventions due to the high degree of co-morbidity between these disorders in primary care and the similarity in epidemiological profiles and treatment responsiveness [1,3-5]. In South-East Asia, 11% of Disability Adjusted Life Years and 27% of Years Lived with Disability are attributed to neuropsychiatric disease[6]. Depression is the most important neuropsychiatric cause of disease burden [7] and CMD lead to profound levels of disability through symptoms such as tiredness and sleep problems, and are associated with increased health care costs and reduced economic productivity [8-11].

The majority of persons with CMD in developing countries seek health care in primary care [7]. A review of 8 epidemiological studies of CMD in South Asia [12] showed prevalence in primary care of 26.3% (95%CI 25.3%–27.4%). Recognition is poor in primary care, with fewer than 1/3^rd ^of clinically significant morbidity detected[13]. Primary care doctors tend to prescribe a greater number of symptomatic medications, such as injectable vitamins, for patients with CMD [14]. A WHO multinational study in general health care reported that nearly 10% of primary care attenders with CMD in the Indian study centre were prescribed psychotropic drugs[15]; however, the majority of prescriptions were for tranquilizers (benzodiazepines) rather than antidepressant drugs.

Three randomised controlled trials studying the efficacy of treatments for CMD in India, Uganda, and Chile were published recently [16-19] All the studies targeted poor populations and tested treatment options that were intended to be feasible, affordable, and acceptable to the populations being studied. These trials attested to the efficacy of antidepressants and brief psychological treatments (interpersonal therapy), delivered in a stepped care model. All the trials showed significant improvements in disability levels in the intervention group; the Indian trial also showed that treating CMD produces significant reduction in total health care costs while the Chilean trial found that the additional cost for each depression-free day was roughly equivalent to the cost of a single bus fare [20].

Although evidence of the efficacy of antidepressants and brief psychological treatments has been available for decades[21], the scaling up of this evidence to routine primary care has been challenging [22-24]. Efforts to improve the primary care treatment of CMD in developed countries include the development of treatment guidelines for CMD and dissemination of guidelines via the education of primary care providers[25,26]. Physician education has been found to be associated with an increase in the recognition of CMD, but not with lasting improvements in patients' clinical outcomes [22,25]. Successful quality improvement programs in developed countries have included, in addition to improving recognition rates, strategies which address other challenges such as the chronic and recurrent nature of CMD and the low adherence with evidence based treatments [27-31]. A recent systematic review of the constituents of complex, collaborative care interventions which improve effectiveness for CMD [32] found that the use of routine screening of all attenders and the professional background of staff and specialist supervision predicted a favourable outcome.

However, this effectiveness evidence cannot be readily transferred to developing countries due to the widely different health systems. There are a number of obstacles to scaling up efficacious interventions to the 'real-world' primary care context in developing countries [33-35]. The first is the low recognition rate of CMD by primary care doctors, for example because of somatic presentations [36]. The second is the inadequate use of evidence-based medications, including antidepressants (at inadequate dosage or for too short durations), and the frequent use of non evidence-based medications [37]. The third obstacle is that few patients receive non-drug treatments for CMD, typically because of the lack of human resources for psychosocial treatments. There is also a severe shortage of skilled mental health resources [38], and finally, low adherence with treatments. Although training programmes for health workers often show an increase in knowledge, the improvement in recognition rates are transient [36], and translation to improved clinical outcomes has not been evaluated [34,39]. Evidence from mental health care demonstration projects show that expansion of care to within reach of the majority of the population in developing countries will necessitate appropriately trained non-specialized health workers working with specialized personnel [34]. The integration of mental health in primary care is acknowledged as the only feasible way of managing the burden of CMD in developing countries [7] yet there is no evidence that such care can be implemented effectively [34]. However, an intervention which seeks to achieve this goal will need to consider strengthening the human resources and support for primary care practitioners, which will inevitably entail increased financial investment. The potential that the intervention will be scaled up may thus hinge on its cost-effectiveness.

## Design and Methods

### Aims

We hypothesize that an intervention strategy based on a Collaborative Stepped Care model, will be clinically- and cost-effective, compared to Enhanced usual care control, for the treatment of CMD and Depression in primary care attenders. The hypothesis concerning cost-effectiveness is important since the former intervention involves additional human resource inputs in a resource restricted environment.

### Objectives

The primary objective is to evaluate the impact of the intervention on the proportion of subjects who recover from an ICD10 defined CMD at 6 months follow-up. Secondary objectives include evaluating the impact of the intervention among the sub-group of those who are suffering from a Depressive Episode at baseline, the mean CIS-R score among those who are probable (i.e. screen-positive) cases at baseline, and the prevalence of ICD-10 CMD at 6 months among those who were sub-threshold cases at baseline.

### Setting

The study is being conducted in Goa, a state in west India with a population of 1.4 million. Goa has been the setting of studies on the epidemiology and treatment of CMD for eight years [16,40-45]. It is estimated that at least 50% of primary care in India (and in Goa) is delivered in the private sector [46] and about half the population lives in rural areas [47].

### Study design

The stepped care model will be evaluated using a cluster-randomized trial which will be implemented in two consecutive phases over 2 1/2 years beginning in April 2007. Phase 1 involves 12 public facilities (Primary Health Centres or PHCs), 6 of which have been chosen at random to deliver the stepped care model to patients screened positive for CMD with the other 6 "control" facilities delivering enhanced usual care control. Phase 2 will be conducted in 12 private facilities (General Practitioners or GPs).

### Selection of facilities/randomisation

The sampling frame for phase 1 consisted of PHCs with minimum space available for the intervention team and which were not involved in preliminary phases related to the intervention development, and which have at least 350 attenders per month. Facilities were stratified into three strata; urban with a visiting psychiatrist (VP), rural with a VP, rural without a VP. Two intervention and two control PHCs were selected at random from each stratum, using on-line software by the MANAS trial statistician (HW) [48]. A given seed number was used to enable the randomization procedure to be reproduced. This guards against misallocation or changes in allocation at a later stage. The sampling frame for phase 2 will consist of all GPs with adequate clinic space and who consent to participate, and will be similarly stratified.

#### The interventions

##### The collaborative stepped care (CSC) intervention

The model for the CSC intervention is based on the stepped-care approach used in the Chilean trial [18] which is ideally suited for treating CMD where a range of severity is likely to be encountered. This approach emphasizes that while simple interventions such as recognition and psycho-education may be provided to all participants, more resource-intensive interventions such as antidepressants and psychotherapy may be reserved for participants who are severely ill or not responding to the simple interventions. Thus, the approach maximizes the efficient use of health resources. Two general principles guide the choice of intervention components: the use of evidence based treatments for CMD; and the collaborations between primary care medical and non-medical staff, and between primary care and specialist staff. The intervention consists of four key stages (recognition; treatment initiation; follow-up and outcomes monitoring, and referral for expert consultation if needed). These stages address specific obstacles to the integration of mental health in primary care. Examples of the specific components for each stage of the intervention are presented in Table 1. The steps in which they will be delivered are described in Table 2.

**Table 1:** Detailed steps of the collaborative stepped care intervention

Step	For whom	Timing	Treatment	BY WHOM
Recognition	Adult PHC patients	Before consultation with PHC doctor	Screening questionnaire; report for doctor	Health Assistant
1	Patients screened with CMD	At first consultation	Advice regarding screening questionnaire results; advice regarding seeing HC Psychoeducation; follow up appointment within 2 weeks. Yoga	PHC doctor HC HC
2	Patients who are severely ill at first consultation, or whose symptoms persist at follow-up	At first consultation or at follow up if not responding to Step 1	Antidepressants OR start IPT in case the patient does not want Antidepressants & Adherence Management	PHC doctor HC HC
3	For participants who remain unwell, or are not adherent	Patients who do not respond to Step 2 despite taking the treatment	Antidepressants & IPT & Adherence Management	PHC doctor HC HC
4	For participants who do not respond despite good adherence	Patients who do not respond to Step 3 despite taking the treatment & Patients who are expressing suicidal ideas at any time	Continue all existing treatments Refer to Clinical Specialist	HC & PHC doctor Clinical Specialist

**Table 2:** Analysis groups

**Group**	**Definition**	**Determined by**	**Rationale**	**Primary Outcome/s**
Possible Cases	Screen Positive CMD*	GHQ12 score	Feasible if rolled out and of significance to public health and primary care professionals – but includes 30% false positives;	Prevalence of ICD10 defined CMD* at 6 months (primary) Mean scores on CIS-R (secondary)
Sub-Threshold Cases	Screen Positive CMD*, but not ICD10 cases	CISR computer algorithm	Sub-threshold cases who are at high risk of developing case-level CMD	Incidence of ICD10 CMD* in 6 months (secondary)
Definite Cases	ICD 10 defined CMD*	CISR computer algorithm	Narrower, biomedical category of significance to mental health professionals	Prevalence of ICD10 defined CMD* at 6 months (secondary)
Depression Cases	ICD 10 defined depression	CISR computer algorithm	Narrower, severe biomedical category of significance to mental health professionals	Prevalence of ICD10 defined CMD at 6 months (secondary)

*(including anxiety disorders, mixed anxiety-depressive disorder, and Depression)

Screening is an essential component of our intervention, ensuring relatively rapid recognition of CMD in this setting where efforts to improve recognition through training of doctors has not yielded sustainable improvement. Psycho-education focuses on educating the person about their symptoms, their association with CMD, the association of CMD with personal difficulties, the need to share emotional symptoms with the doctor and to share personal difficulties with caring family members or other key persons in their social network. Psycho-education teaches patients simple strategies for symptom alleviation. Our choice of inter-personal therapy as the psychological treatment is based both on its demonstrated feasibility and effectiveness in another developing country [49], and on its focus on interpersonal problems such as grief, disputes and role transitions, which were consistent themes in the adverse life experiences of participants in the earlier research in Goa [50]. A minimum of 6 sessions, with an optimum of 8 and a maximum of 12 sessions, will be offered to each eligible participant. The choice of antidepressant is based on our earlier research which showed better tolerance of low-cost SSRI (selective serotonin reuptake inhibitors) such as fluoxetine [51], which was also the antidepressant used in the previous Goa trial. Fluoxetine is cheap in India, and has recently come off patent globally. However, fluoxetine is not available in PHCs and will therefore be provided by the project (to integrate with the existing model of free medicines prescribed by the PHC doctor). In the private GP phase, however, doctors will be free to use other antidepressants which will need to be purchased by patients as they would do with any other medicines prescribed by the GP. For both antidepressants and psychotherapy, we have set *a priori *criteria for "minimum adequate" treatment. For antidepressants, this criterion is use for at least 90 days at a minimally adequate dose (at least 20 mg per day of fluoxetine or the equivalent), and for IPT, attendance at least six sessions.

The intervention was developed in a systematic three-stage preparatory phase over 15 months, which has been described in detail previously [52]. The intervention will be delivered by four key persons in each facility: a Health Assistant, a Health Counselor (HC); the primary care doctor/s; and a psychiatrist in the role of Clinical Specialist. The Health Assistant will screen adult patients attending the PHC using the 12 item General Health Questionnaire (GHQ) [53] to identify those who are suffering from a CMD and report the results to the PHC doctor. The choice of the GHQ was based on a study evaluating five possible screening questionnaires carried out as preparation for the trial [54]; the GHQ was found to have the highest discriminating properties, both for CMD as a broad clinical category and for the narrower category of Depressive Episode.

The HC will lead the intervention. She will be a locally recruited graduate non-medical worker who will take overall responsibility for the intervention in close collaboration with the primary care doctor; she will deliver all the non-drug treatments. The principal role of the primary care doctors will be to initiate antidepressant treatments and provide usual care for any co-existing physical health problems. Each facility team will be supported by a Clinical Specialist, a psychiatrist who will act as a specialist resource person to train and support the primary care doctors and the HCs. Details of the intervention can be found in Table 3 and in the publication describing its development [52].

##### The enhanced usual care control (EUC) intervention

In order to assemble comparable samples of patients in the intervention and control practices, it is necessary to conduct systematic screening in both groups. If we are screening in the usual care practices, it is unethical to conceal screening results from treating physicians. Consequently, physicians in usual care practices will receive screening results and may choose to initiate treatment. This enhancement to usual care in the control practices may introduce a conservative bias, i.e. reducing the difference in outcomes between intervention and control practices, but it is ethically necessary. This will be introduced in control facilities. They will have only one additional staff, viz. the Health Assistant, who will screen patients for CMD, and fluoxetine will be provided by the project to PHCs but not to private GP practices, as in the Collaborative Stepped Care arm.

We do not anticipate a significant risk of contamination, i.e. patients moving from an Enhanced usual care control facility to an intervention facility, due to the geographical spread of facilities, and because no publicity will be produced regarding the availability of the intervention in other facilities. In addition, we do not anticipate a significant therapeutic effect in the Enhanced usual care control arm given that screening and recognition, or the provision of evidence based guidelines, are not, by themselves, sufficient to lead to clinical improvements [22,25].

### Selection of study participants

**Figure 1:**
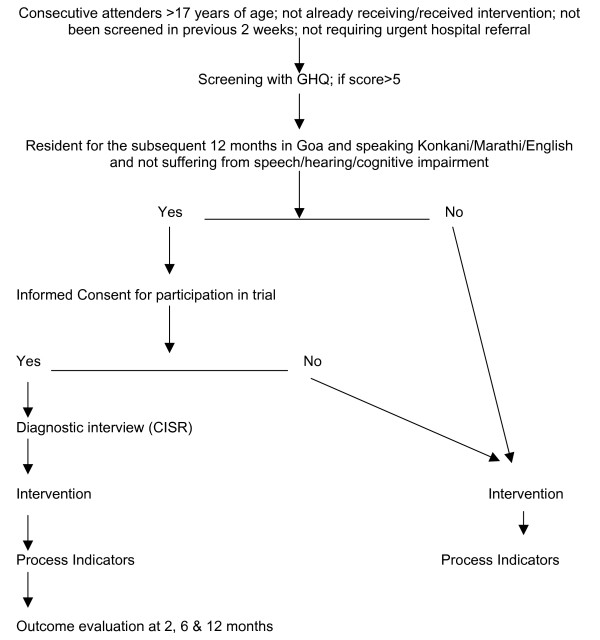
Trial Flow Chart: selection of participants in each facility.

The flowchart for selection of trial participants is shown in Figure 1. Eligibility criteria for screening by the Health Assistant are: age >17 years, not requiring urgent medical attention, not already screened in the previous 2 weeks; and not already receiving the intervention. All patients who screen positive (GHQ score >5) will be notified to the GP, and eligible to receive whichever intervention programme is being delivered in the facility. Those who, in addition, fulfill the following criteria will be invited to participate in the outcome evaluation of the trial: resident in Goa for the subsequent 12 months; speak one of the three primary study languages (Konkani, Marathi, English); and do not suffer from a serious impairment (hearing, speech, cognition) which interferes with participation in an interview. If the patient gives written or verbal consent, the Health Assistant will carry out a structured clinical diagnostic interview (the Revised Clinical Interview Schedule or CIS-R) [55] which has been previously field tested in Goa [56].

### Trial objectives and outcomes

It is our objective to study the overall effect of the intervention on the treatment and outcome of CMD in primary care. Four primary research questions are being addressed in the trial, relating to clinical outcomes in different groups of patients, defined according to their type of CMD at baseline (Table 2). From a practical or policy perspective, we need to evaluate the impact on all patients who are identified on the basis of the short screening procedure. Our primary analysis will focus on the subgroup for whom previous evidence is clearest regarding benefit: definite ICD-10 CMD. The outcome will be assessed through home visits at 6 months using the CISR which generates both CMD case and Depression case outcomes based on ICD10 criteria; and a CISR total score. Six months is chosen as the primary endpoint because it marks the maximum duration of the first-line drug and psychological components of the intervention. Other end-points at 2 and 12 months will assess the rate and persistence of clinical recovery. Other outcomes include recovery from Depression, the mean CIS-R score, and prevalence of ICD-10 among sub-threshold cases (Table 2).

In addition, economic costs and outcomes will be measured using the Costs of Illness Schedule, developed for economic analyses of mental disorders in India [9] and used in the previous efficacy trial in Goa [16]. This schedule includes both direct and indirect costs, due to health problems, incurred by the participants, but does not include the direct costs of the intervention itself. The latter will be computed by deriving a monetary value for each component of the intervention based on actual costs, and applying these to each individual based on the process indicators which reflect the actual contact with the intervention. Disability levels will be assessed using the WHO12 item Disability Assessment Schedule, based on the Brief Disability Questionnaire which has been used in Goa [14,57], and input into the estimation of opportunity costs.

### Outcome evaluation/masking

Outcomes will be assessed through home visits by a team of researchers independent of the intervention team and blind to the allocation status of the patient. The primary end-point assessment will occur within a month of the 6 month follow-up date; similarly for the 2 and 12 month assessments. Masking will be maximized by:

• Randomly allocating unique patient IDs so that there is no association between the ID number and the facility identity.

• Ensuring that the outcome evaluation is carried out by an independent institution whose team is not privy to the randomization allocation.

• Emphasizing to assessors that all patients are receiving an intervention (not specifying whether this is enhanced care or Collaborative Stepped Care) and that there is genuine equipoise about which is better

• Carrying out CISR (the primary outcome) assessments prior to all other outcome assessments.

• Assessing the efficacy of blinding (through asking assessors to guess which arm the participant is allocated to) at the end of the trial

• Ensuring that process and qualitative research evaluations (not described in this protocol) are carried out independently of the quantitative evaluations; the qualitative research will be carried out only with participants who have consented, but who are not selected for the quantitative evaluation.

• Inserting a 'dummy' question at the 6 month follow-up which assesses a health state (fever) which is not influenced by the intervention.

#### Sample size

##### a) Definite cases

150 participants with a GHQ score >5 will be recruited from each of 24 facilities. Based on the piloting stage experience that up to 25% of these participants will either not be traced by the outcome evaluators or will not consent to participate, and that at least 1 in 3 'cases' identified by the GHQ are Sub-Threshold cases, we anticipate that at least 75 definite cases (see Table 3) from each facility will complete the outcome evaluation at the 6 month end-point. This gives a total sample size of 1800 in which to assess our primary outcome: recovery rate of ICD-10 defined CMD at 6 months among definite cases at baseline. This will have more than 90% power to detect at the 5% level of significance a difference in recovery rates of 70% in the Collaborative Stepped Care versus 50% in the Enhanced usual care control arm. If the recovery rates are 65% and 50% respectively, we will have 74% power to detect a significant difference. Similarly, we have around 90% power to detect a difference in proportion recovered from depression at 6 months of 50% in the Collaborative Stepped Care arm versus 30% in the Enhanced usual care arm.

The assumptions underlying this calculation are as follows. The recovery rates in the Chilean trial [18], whose intervention model is the one being used in this study, showed a gap in recovery at 6 months of 70% versus 30% between the two groups. However, our trial is evaluating effectiveness of introducing this model into routine care, and so we would expect a smaller difference between rates than in an efficacy trial. Furthermore the Chilean trial focused on Depression, while ours will also recruit subjects with less severe CMD who might potentially have higher spontaneous recovery rates in both treatment arms. There are no existing data on the recovery rates of CMD in routine PHC or GP in India. The limited data from other developing countries, including outcomes of Usual Care groups in efficacy trials, suggests that between 30% and 50% of subjects with a CMD recover without active intervention by 6 or 12 months after recruitment [16,18,58]. We therefore based our primary sample size calculation on a likely conservative difference of 20% between the two arms (compared to 40% in the Chile trial), and on recovery rates of 30–50% in the Enhanced usual care control arm.

A key factor in estimation of the sample size for a cluster RCT is the coefficient of variation (SD/mean) of the true proportions with the outcome between clusters within each arm [59]. In the absence of outcome data to estimate k in Goa, we have used the recovery rates of CMD in the two Asian centres (Shanghai, Bangalore) of the WHO multinational study of CMD in general health care [10], and data from 8 studies of CMD prevalence in India (two in urban GPs, three in urban PHCs and three in rural PHCs) [12] all of which estimate k < 0.2. The proposed trial is located in one state of India and facilities are stratified according to their rural/urban location and private/public sector; hence the value of k is likely to be even lower [59,60]. To be conservative, we have used an estimate of k = 0.2.

##### b) Possible cases

We expect about one-third of possible cases to be sub-threshold cases at baseline, as defined by the CIS-R, and that 10% of these might become new cases in the EUC arm compared to 5% in the CSC arm. In addition, we assume that 50% of the definite cases at baseline will still be cases at 6 months in the control arm, compared to 30% in the intervention arm. So overall, we will expect prevalence of CMD at 6 months amongst possible cases at baseline to be 37% in the control arm (10% × 1/3^rd ^+ 50% × 2/3^rd^), and 22% in the intervention arm (5% × 1/3^rd ^+ 50% × 2/3^rd^). We will have 99% power to detect this difference at the 5% significance level, assuming a 75% follow-up rate as above.

A secondary outcome in this group is the mean CIS-R score at 6-months among probable, i.e. screen-positive, cases at baseline. Our earlier longitudinal study of confirmed ICD10 cases at baseline found a reduction in mean CISR score of 50% over 6 months from 24 (s.d. 6) to 12 (s.d. 10) [16]. We would expect slightly lower CISR scores among all possible cases at baseline in our study. Our sample size gives high power (100%) to detect a difference in CIS-R score at 6 months of 18 (s.d. 10) in the Enhanced usual care control arm versus a CIS-R score of 9 (s.d. 10) in the Collaborative Stepped Care arm.

##### c) Sub-threshold cases

The only cohort study of primary care attenders which has reported on risk factors for new cases of CMD from a developing country was carried out in Zimbabwe [61]; in this study, the proportion of sub-threshold cases at baseline who became cases at 6 months was 31% (19 out of 61 subjects). Assuming a 75% follow-up rate we will have an effective sample size of 900 sub-threshold cases; this gives about 70% power to detect a difference in the proportion with CMD at 6 month follow-up between 5% in the intervention arm and 10% in the control arm.

##### d) Depression cases

Our pilot data has shown that only 1/3 of CMD cases are suffering from ICD-10 depression. Thus the proposed sample size would yield about 25 depression cases per facility traced at 6 months, giving a total effective sample size of 600; this has 94% power to detect a difference in recovery rates of 30% in the Enhanced usual care control arm and 50% in the Collaborative Stepped Care arm at 6 months. Again these are conservative estimates compared to the achievements of the Chile trial.

##### e) Cost effectiveness

We have not powered our trial for the cost-effectiveness outcome because we expect the intervention to be dominant as compared to the usual care, i.e. improved outcomes *and *lower costs. Thus, additional resources required to implement the intervention are expected to be fully or partially offset by reduced consumption and associated costs of other health care services (especially outpatient attendance for somatic symptoms) and to also lead to reduced opportunity costs to patients and families (especially lost work days). This is what we found for the antidepressant arm in the earlier cost-effectiveness trial in Goa [16].

### Data management & analyses

Data will be collected using a hand-held computer (Palm-OS), and exported to Stata 9.0 for statistical analysis. Given direct entry of responses, we expect there to be minimal data entry errors. Findings will be reported as per the CONSORT guidelines for cluster randomized controlled trials [62]. Baseline comparability will be assessed for individuals who did not consent to be part of the trial, and of participants who could and could not complete review assessments. Comparability of the two intervention arms will be assessed for a variety of potential confounding factors assessed at 2 months, notably: age, sex, education, per capita income, severity of mental health scores, ICD10 diagnostic distribution, and comorbidity with alcohol use and physical health problems.

The primary analyses will be intention-to-treat, regardless of adherence to the intervention, and will be based on outcomes 6 months after diagnosis. Logistic regression generalized estimating equations with robust standard errors will be used to compare case prevalence in the two arms, allowing for any within-facility clustering resulting from the cluster randomized design [63], and adjusting for baseline mental health score. Further analyses will adjust for any other of the a-priori defined confounding factors list above for which randomization did not achieve balance between the two arms at baseline. These analyses will be carried out for the different patient groups defined in Table 3, with their respective outcomes as described above. For each of these primary outcome analyses, we will present an estimate of the effect size as an odds ratio and 95% confidence intervals, and a coefficient of intracluster correlation. A number of secondary analyses are proposed, which are listed in the Trial Registration protocol.

For the economic analysis, health care costs and other patient- or family-borne costs will be computed and compared at 2, 6 and 12 months, and subsequently related to changes in health outcome: both the primary outcome measure of depression status and also Quality Adjusted Life Years (QALYs). A culturally specific and conceptually solid utility measure for depression – necessary for the estimation of QALYs – will be generated by relating health state valuation scores (taken from an Indian population as part of a wider WHO multi-country study) to summary scores on the WHO-DAS. In the event that dominance is not shown, i.e. the intervention is more effective but the costs are also more than the usual care group, we will estimate the probability of dominance and estimate incremental net benefit and incremental cost-effectiveness ratios. The latter will be computed, together with their confidence intervals (using bootstrapping techniques to overcome expected skewness of cost data). Cost-effectiveness acceptability curves will also be derived in order to show the probability of any cost-effective advantages for the component interventions at a range of 'willingness to pay' threshold levels.

### Trial monitoring

The following 'sentinel' outcome data will be recorded upon completion of interviews for monitoring and safety requirements, and reported to the Trial Steering Committee and Data Monitoring & Ethics Committees at least quarterly: number and proportion of completed outcome assessments and refusals; ICD10 case status for any CMD and Depression; hospitalizations; suicide attempt; and deaths.

No stopping rules are proposed because serious adverse events are not expected in the trial since none of the treatments being offered are experimental or associated with serious outcomes. A recent systematic review has shown that RCTs stopped early show implausibly large treatment effects and that there needs to be a balance between the ethical concerns of safeguarding interests of patients who have been randomized in the trial while also "protecting society from overzealous premature claims of treatment benefit [64,65]." Furthermore, the trial will proceed to phase 2 (GP facilities) without conducting formal interim analyses at the end of phase 1 (PHC facilities) since, even if such analyses showed considerable impacts of the intervention, these may not be generalizable from PHC to GP facilities.

## Ethical considerations

While the efficacy of specific intervention components (e.g., anti-depressant drugs) is established in developing countries, the same is not true for the effectiveness of intervention strategies that attempt to introduce these into usual primary care. The proposed trial is addressing whether a collaborative stepped-care intervention enables these treatments to be provided effectively in primary care and, if so, what are the marginal benefits and costs. There is genuine clinical equipoise concerning this research question in the context of developing countries where the intervention presents additional costs and where there are several other public health priorities. Under-powered trials are unethical for patients [66]; our sample size calculations are therefore based on conservative estimates and high power. No participant will be deprived of any treatment s/he would ordinarily receive. Participants in the enhanced usual care control facilities may benefit as a consequence of the screening and, in the PHCs, the provision of antidepressants to the pharmacy. Thus they will be provided with the results of the screening questionnaire and those who remain ill at 12 months will be offered psychiatric consultation by the Clinical Specialist. In addition, if any trial participant (in either arm) is found to have attempted suicide (assessed during outcome evaluation), the field work team will report this through an administrator to the intervention team who will make arrangements to provide psychiatric care for the participant. Explicit referral guidelines will be provided to doctors in the Enhanced usual care control arm for patients who may be suicidal or need specialist advice (to the local public psychiatric services available in three hospitals in Goa).

In cluster RCTs, consent for participation in a trial is needed both from the clusters and, depending on the type of intervention being delivered and outcomes assessed, from individual participants. In the MANAS trial, cluster level consent is being obtained as follows:

• Phase 1: PHC consent has been obtained in two steps; first, the Government of Goa's Directorate of Health Services; after the final 12 PHCs were identified and randomized, specific written consent was obtained from the DHS for the participation of these 12 PHCs.

• Phase 2: Individual facility level consent from each GP is implicit since only those who are consenting to participate will be included in the sampling frame.

Individual participant consent is being obtained in two stages: after the screening, those who are screen-positives will be invited to participate in the CIS-R interview and thereby be enrolled in the trial; and for those selected for outcome evaluation, formal written consent will be obtained by the field researcher at the first visit to the participant in his/her home 2 months after enrollment.

Formal ethical approval has been obtained from the IRBs of the lead Goan organization (Sangath) and the London School of Hygiene & Tropical Medicine and has been approved by the Indian Council for Medical Research. The Trial Steering and Data Monitoring & Ethics Committees will monitor the progress of the trial. A fresh report will be made to both ethics committees of major changes in the protocol since the original proposal was assessed.

## Discussion

While there is evidence on *what *treatments work for CMD in developing countries, the question of *how *these can be effectively delivered in routine primary care remains unanswered [67]. This is one of the major research priorities in global mental health [68]. The results of the trial will be used to inform policy makers and practitioners in developing countries on the practical implementation, and the clinical and economic benefits of improving the management of CMD in primary care. The trial is likely to yield findings which are generalisable beyond the population of the study and, if effective, could be scaled up in health policy due to: the representative sampling of participants from rural and urban, private and public primary care facilities; the use of an intervention strategy which integrates evidence from efficacy trials from three developing countries and emphasizes acceptability (to practitioners, policy makers and participants), affordability (acceptable cost-effectiveness ratio) and availability (use of locally available resources); and the application of methods to address obstacles to the integration of mental health in primary care.

We acknowledge that the trial intervention will not tackle all the obstacles to the integration of mental health in primary care, such as the stigma associated with mental illness in the community [33,35]. Some components of the intervention may not seem generalisable. First, is screening a feasible component of the intervention? Recognition of CMD is an essential requirement for providing an intervention. A strictly naturalistic trial may have chosen to recruit subjects referred by the primary care doctor. We ruled this out since there is good evidence to show that recognition rates are very low [10,12,14,56] and that training doctors does not lead to sustainable higher rates of recognition[33]. Furthermore, a recent systematic review of collaborative care interventions found systematic identification of patients was one of only three specific ingredients which predicted a favorable response to the intervention [32]. Thus, screening in this trial is an integral component of the intervention as well as being used to identify a comparable sample of CMD patients in the Enhanced usual care control arm. With regards to scaling up, we believe that screening is feasible because of the high prevalence of CMDs in primary care attenders, the brevity of screening instruments (which may be reduced to just a couple of questions) [69] and the increasing literacy rates in many countries which makes self-completion feasible. The second component is that of the need for employment of a Health Assistant and a Health Counselor, additional human resources, in primary care facilities. However, the type of persons we will use in this role is relatively low-cost. Such persons who might suit these roles are easily available in most developing countries and could perform other roles, such as behaviour modification and lifestyle interventions for other chronic diseases. If Ministries of Health are to consider supporting mental health programmes which involve additional resources, data on the feasibility and cost-effectiveness of interventions are critical [34]; the proposed trial will provide the first systematic evidence of this kind from a low income country.

## Competing interests

The author(s) declare that they have no competing interests.

## Authors' contributions

VP is the PI of the trial, wrote the original and revised protocol and the draft of this paper;

BK made substantive contributions to the study design, was involved in drafting the manuscript and approved the final submitted version;

SP is the research coordinator of the project, and made contributions to the study design during piloting, was involved in drafting the manuscript and approved the final submitted version;

RA made substantive contributions to the study design, was involved in drafting the manuscript and approved the final submitted version;

MK made substantive contributions to the study design, was involved in drafting the manuscript and approved the final submitted version;

DC is the health economist who designed all the economic components of the trial; he was also involved in drafting these sections of the manuscript and approved the final submitted version;

GS made substantive contributions to the study design, was involved in drafting the manuscript and approved the final submitted version;

HW is the trial statistician; she made substantive contributions to the study design, was involved in drafting the manuscript and approved the final submitted version.
